# Aerobic exercise-induced HIF-1α upregulation in heart failure: exploring potential impacts on MCT1 and MPC1 regulation

**DOI:** 10.1186/s10020-024-00854-3

**Published:** 2024-06-12

**Authors:** Longfei Xu, Miaomiao Yang, Aili Wei, Zilin Wei, Yingkai Qin, Kun Wang, Bin Li, Kang Chen, Chen Liu, Chao Li, Tianhui Wang

**Affiliations:** 1 Military Medical Sciences Academy, Tianjin, 300050 China; 2 Tianjin Key Laboratory of Exercise Physiology & Sports Medicine, Tianjin University of Sport, Tianjin, 301617 China; 3 No. 950 Hospital of the Chinese People’s Liberation Army, Yecheng, 844999 China

**Keywords:** Aerobic exercise training, Heart failure, HIF-1α, MCT1, MPC1, Hypoxia

## Abstract

**Background:**

The terminal stage of ischemic heart disease develops into heart failure (HF), which is characterized by hypoxia and metabolic disturbances in cardiomyocytes. The hypoxic failing heart triggers hypoxia-inducible factor-1α (HIF-1α) actions in the cells sensitized to hypoxia and induces metabolic adaptation by accumulating HIF-1α. Furthermore, soluble monocarboxylic acid transporter protein 1 (MCT1) and mitochondrial pyruvate carrier 1 (MPC1), as key nodes of metabolic adaptation, affect metabolic homeostasis in the failing rat heart. Aerobic exercise training has been reported to retard the progression of HF due to enhancing HIF-1α levels as well as MCT1 expressions, whereas the effects of exercise on MCT1 and MPC1 in HF (hypoxia) remain elusive. This research aimed to investigate the action of exercise associated with MCT1 and MPC1 on HF under hypoxia.

**Methods:**

The experimental rat models are composed of four study groups: sham stented (SHAM), HF sedentary (HF), HF short-term exercise trained (HF-E1), HF long-term exercise trained (HF-E2). HF was initiated via left anterior descending coronary artery ligation, the effects of exercise on the progression of HF were analyzed by ventricular ultrasound (ejection fraction, fractional shortening) and histological staining. The regulatory effects of HIF-1α on cell growth, MCT1 and MPC1 protein expression in hypoxic H9c2 cells were evaluated by HIF-1α activatort/inhibitor treatment and plasmid transfection.

**Results:**

Our results indicate the presence of severe pathological remodelling (as evidenced by deep myocardial fibrosis, increased infarct size and abnormal hypertrophy of the myocardium, etc.) and reduced cardiac function in the failing hearts of rats in the HF group compared to the SHAM group. Treadmill exercise training ameliorated myocardial infarction (MI)-induced cardiac pathological remodelling and enhanced cardiac function in HF exercise group rats, and significantly increased the expression of HIF-1α (*p* < 0.05), MCT1 (*p* < 0.01) and MPC1 (*p* < 0.05) proteins compared to HF group rats. Moreover, pharmacological inhibition of HIF-1α in hypoxic H9c2 cells dramatically downregulated MCT1 and MPC1 protein expression. This phenomenon is consistent with knockdown of HIF-1α at the gene level.

**Conclusion:**

The findings propose that long-term aerobic exercise training, as a non- pharmacological treatment, is efficient enough to debilitate the disease process, improve the pathological phenotype, and reinstate cardiac function in HF rats. This benefit is most likely due to activation of myocardial HIF-1α and upregulation of MCT1 and MPC1.

**Supplementary Information:**

The online version contains supplementary material available at 10.1186/s10020-024-00854-3.

## Introduction

Heart failure (HF) is a widespread global public health problem and the leading cause of mortality, which manifests a reduction in the capacity of the heart to meet the metabolic needs of the whole body (Savarese and Lund 2017; Cordero et al. 2019; Ho et al. 2019). Myocardial infarction (MI), mainly resulting from myocardial ischemia and hypoxia, is the primary etiologic factor in heart failure which leads to myocardial necrosis, and pathological remodeling including cardiomyocyte hypertrophy and myocardial fibrosis (Feriani et al. 2020; Hanif et al. 2017; Rabinovich-Nikitin et al. 2019). Despite the compensatory response of cardiomyocytes can transiently maintain the cardiac contractile function, progressive cardiomyocyte hypertrophy may lead to decompensation and deterioration of cardiac function, ultimately leading to malignant arrhythmias and even HF (Kyhl et al. 2017). The metabolic perturbations provoked by myocardial hypoxia are pivotal in HF progression. In the last years, the attention of HF treatment has turned to intervention in myocardial energy metabolism (Yurista et al. 2021; Beltran et al. 2020). One critical aspect of this strategy is HIF-1α, a pivotal participant of the pathophysiological process in ischemic heart disease and HF, particularly in the course of hypoxia and energy metabolism (Semenza 2014a, b). In reaction to cellular hypoxia, HIF-1α, one part of the hypoxia-inducible factor-1 (HIF-1) complex, raises its expression, influencing the expression of genes related to oxygen delivery and metabolic adjustment (Dales et al. 2010; Semenza 2014a, b; Brahimi-Horn and Pouysségur 2007). The hypoxia tissues of infarction heart have reported that increase of HIF-1α gene expression, and its upregulation causes a reduction in myocardial infarct size and improved cardiac function (Semenza 2011; Zhang et al. 2020; Kido et al. 2005).

Pathological myocardial hypertrophy (MH) involves a shift in cardiac metabolism from fatty acids to glucose (Gibb and Hill 2018); HIF-1α activates genes related to glucose metabolism, adapting to these metabolic changes (Li et al. 2020). To compensate for the tricarboxylic acid cycle (TCA) flux in hypertrophied hearts, this alteration promotes enhanced glycolysis and glucose uptake along with compensatory anaplerosis (Kolwicz and Tian 2011; Tran and Wang 2019; Mirtschink and Krek 2016). Nonetheless, adenosine triphosphate (ATP) production is not increased accordingly due to increased glycolysis, unveiled that glycolysis unlinked from glucose oxidation in the pathological hypertrophy (Sorokina et al. 2007). Lactate is made from glycolytic pyruvate and is also an important energy source for the myocardium (Hui et al. 2017; Sambandam et al. 2002). In HF, there is the uncoupling between lactate production and consumption, disrupting pyruvate-lactate axis, which is a hallmark of MH and HF (Bergman et al. 2009; Fillmore et al. 2018; Cluntun et al. 2021). Here, the mitochondrial pyruvate carrier (MPC) and Monocarboxylate transporters (MCTs) in the pyruvate-lactate axis are important nodes for cardiac homeostasis and health. Of these, the only one whose identity was confirmed in the rat heart was MCT1, which was abundantly present and strongly expressed in the cardiomyocyte plasma membrane, including the intercalated disk (Halestrap and Price 1999). Increased levels of MCT1 protein were found in the myocardium of a rat model of congestive heart failure, suggesting that lactate is an indispensable energy source for the failing heart, which may be beneficial to the failing heart and cardiomyocytes (Jóhannsson et al. 2001; Evans et al. 2003). In addition, MPC determines that a large portion of pyruvate is reduced to lactate in the cytoplasm, thereby enabling metabolic adaptation during HF (Fernandez-Caggiano and Eaton 2021). Several proofs revealed that MPC1 and MPC2 expression is down-regulated in failing human and mouse hearts and that MPC deficiency in mice causes pathological MH and cardiac dysfunction (Fernandez-Caggiano et al. 2020; Sheeran et al. 2019; McCommis et al. 2020).

Although there have been major developments in HF, there is still a requirement for therapeutics with a higher efficiency (Bauersachs 2021; Bouri et al. 2014). Exercise is a non-pharmacological treatment that could upgrade cardiovascular fitness and diminish the symptoms and death rate of HF (Adams and Schuler 2012; Levine 2014; Belardinelli et al. 2012; Wisløff et al. 2007; O’Connor et al. 2009). Exercise has been elucidated to upregulate HIF-1α (Song et al. 2020) and MCT1 (Jóhannsson et al. 2001) levels in myocardial tissue and improve cardiac function in rats with MI; whereas a gap still exists in the effects of exercise on MCT1 and MPC1 in HF, and whether HIF-1α induces changes in MCT1 and MPC1 in HF during hypoxia is poorly understood. Under this context, the purpose of the present study was to investigate the effects of exercise training on myocardial HIF-1α, MCT1, and MPC1 expression through our established in vivo model of HF in rats. Additional series of in vitro experiments were also conducted in hypoxia-treated H9c2 rat cardiomyoblasts using activators/inhibitors and shRNA as a means to determine the effects and alterations of HIF-1α on MCT1 and MPC1. We speculate that exercise may upregulate the expression of key proteins (MCT1 and MPC1) by increasing myocardial HIF-1α levels in rats, which could contribute to the improvement of HF prognosis.

## Materials and methods

### Animals

Seven-week-old male Wistar rats (weighing approximately 180 g) were purchased from the BEIJING HFK BIOSCIENCE CO., LTD, (Beijing, China). Rats were acclimatized for 1 week before the experiment to adapt to the new surroundings. Rats were housed cages and had access to food and water ad libitum. The facility environment had controlled light (12:12 h light/dark cycle) and constant room temperature (23 ~ 25 °C) under conventional laboratory conditions. Rats were assigned randomly into four experimental groups (*n* = 6–8 for each group): sham sedentary (SHAM), HF sedentary (HF), HF short-term exercise trained (HF-E1) and HF long-term exercise trained (HF-E2). Short-term exercise lasted for 6 weeks (HF-E1) and long-term exercise lasted for 12 weeks (HF-E2) were defined according to the previous study (Guo et al. 2020). All experimental procedures were approved by the Laboratory Animal Welfare Ethics Committee of Military Medical Sciences Academy. All efforts were made to minimize the number and suffering of animals used in these experiments.

### Myocardial infarction-induced HF model

Experimental rat MI models were established by permanent ligation of the left anterior descending coronary artery (LAD) of the heart (Li et al. 2021). Briefly, the rats were anesthetized with sodium pentobarbital (30 mg/kg) intraperitoneally (i.p.), endotracheally intubated, and mechanically ventilated with room air (respiratory rate 60–70 breaths/min, tidal volume 2.5 mL). Left thoracotomy between the fourth and fifth ribs was performed to expose and access the rat heart. LAD was identified approximately 2 mm beneath the left atrial appendage and ligated (descending aorta constricted by a 7 − 0 silk suture tied snugly). After ligation, the lungs were re-inflated, after which the chest (6 − 0 silk suture) and skin (4 − 0 silk suture) were closed. Sham-operated rats underwent the same surgical procedure except that their LAD were merely threaded and not ligated. The trachea was extubated as soon as the animals began to recover from anesthesia, and rats were subsequently placed in a warm box (30 ~ 32 °C) for 1 h. Penicillin (80,000 U/kg) was injected intraperitoneally for 3 consecutive days after surgery to prevent infection.

### Exercise training protocol

**Fig. 1:**
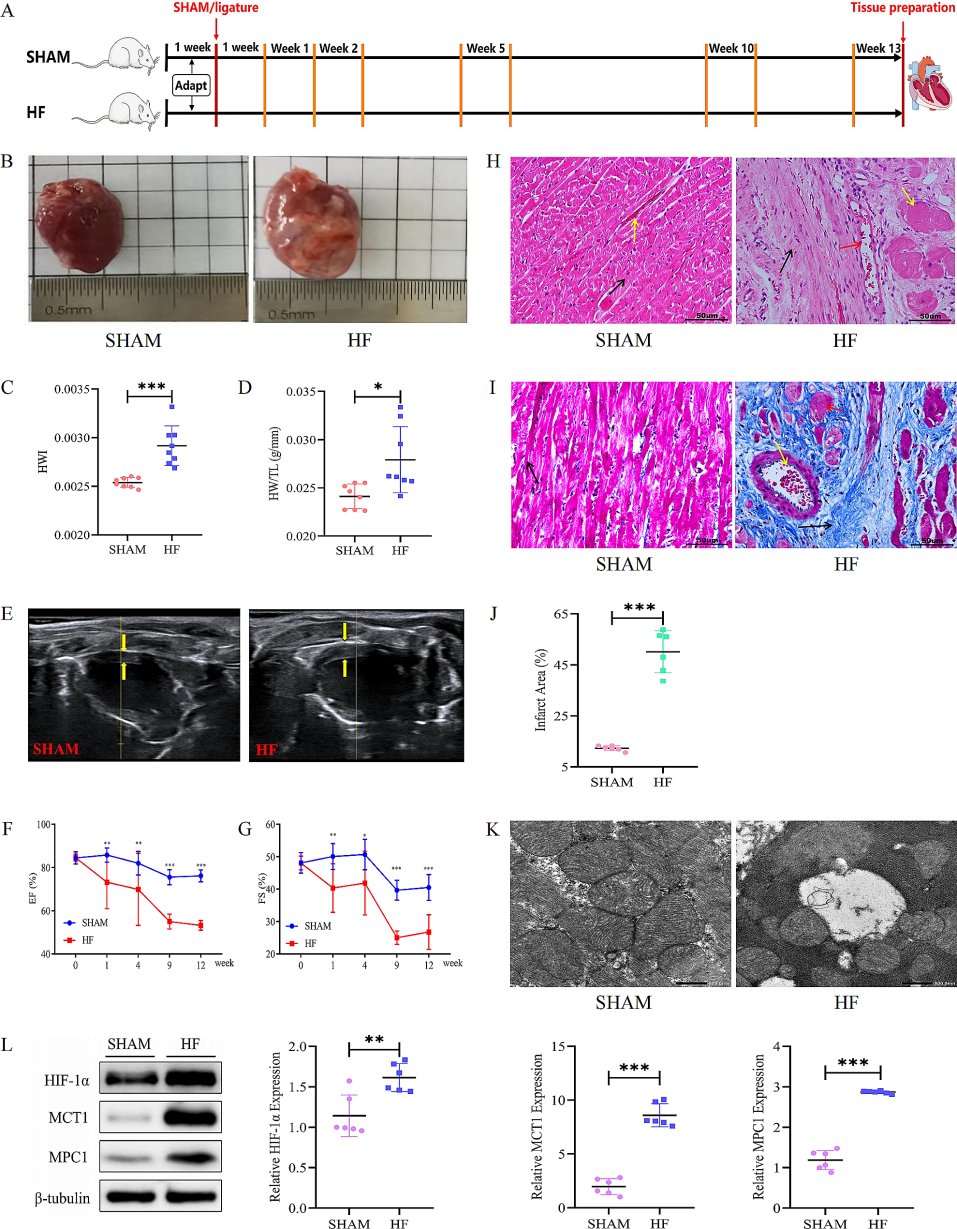
Pathological remodeling in failing hearts post-myocardial infarction. **(A)** The timeline illustrates key moments for evaluating left ventricular function in rat groups via echocardiography. (**B**) Visual comparison of heart morphology across different groups. (**C, D**) Graphs illustrating changes in heart weight/body weight ratio (HWI) and heart weight/tibia length ratio (HW/TL) among the groups. (**E**) Echocardiographic images displaying left ventricular function, with wall thickness indicated between yellow arrows. (**F, G**) Graphical representation of changes in left ventricular ejection fraction (EF) and fractional shortening (FS) before and after LAD surgery Notation ‘*’: denotes statistical comparison between HF and SHAM groups. (**H**) HE staining images showcasing myocardial tissue in various groups. (**I**) Masson’s trichrome staining images display myocardial fibrosis in different groups. (**J**) Quantification of infarct areas based on Masson’s trichrome staining across groups. (**K**) Electron micrographs representing cardiomyocyte ultrastructure among the groups. (**L**) Western blot analysis shows the expression of HIF-1α, MCT1, andMPC1 proteins in myocardial tissues, with tubulin as the protein loading control. The student *t*-test was used for comparisons in the study. Mean ± SD. ^*^: *P* < 0.05, ^**^: *P* < 0.01, ^***^: *P* < 0.001. *n* = 6–8

**Fig. 2:**
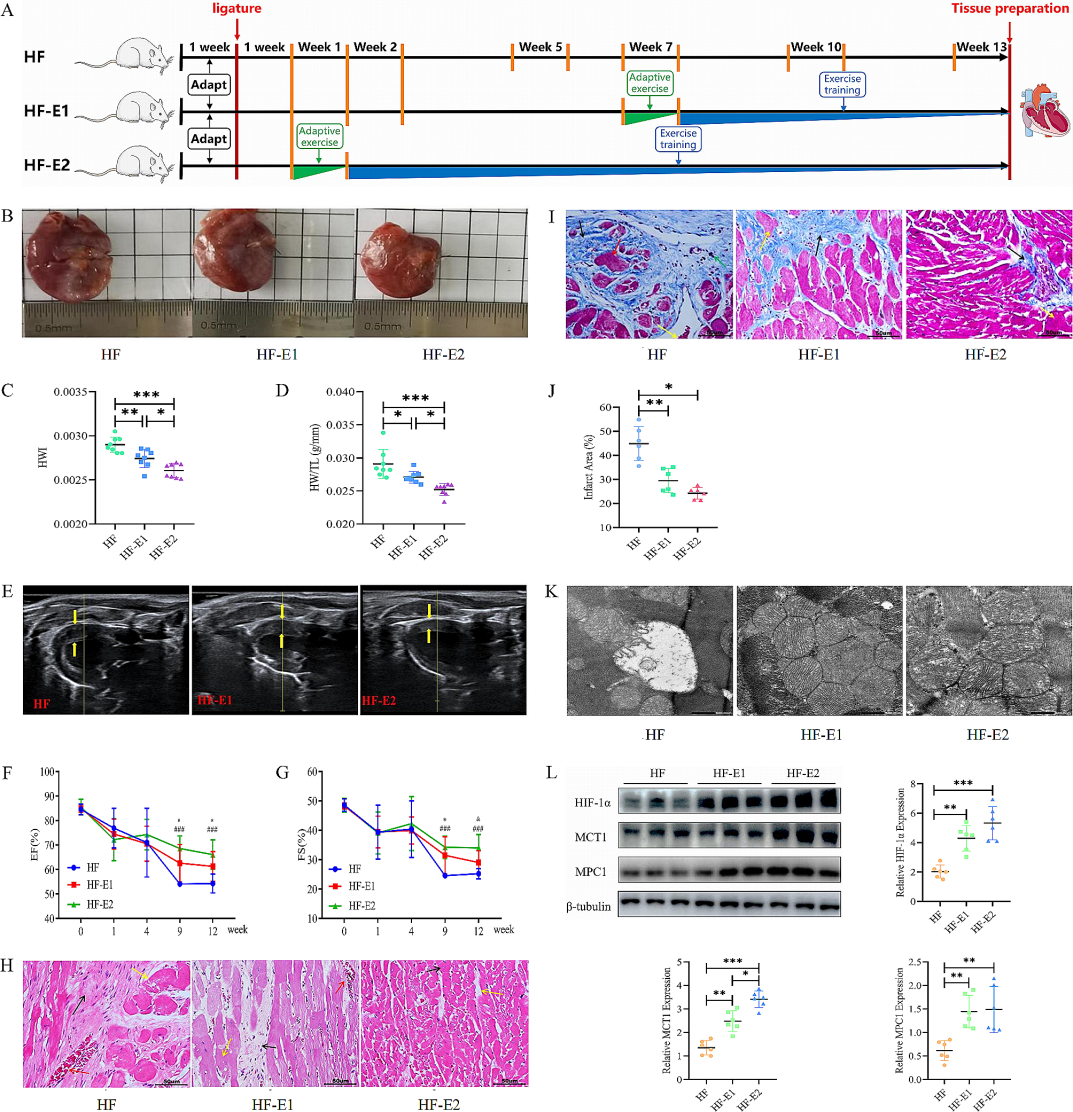
Treadmill exercise training improves pathologic features of the failing heart. **(A)** The timeline displays key points for left ventricular function evaluation using echocardiography in each group, conducted before the LAD artery procedure and subsequently during the 2nd, 5th, 10th, and 13th weeks post-LAD procedure. (**B**) Comparative visualization of heart morphology across groups. (**C, D**) Graphs depicting changes in heart weight/body weight ratio (HWI)and heart weight/tibia length ratio (HW/TL) among groups. (**E**) Echocardiographic images illustrating left ventricular function, with wall thickness highlighted between yellow arrows. (**F, G**) Graphs showing alterations in left ventricular ejection fraction (EF) and fractional shortening (FS) pre and post-LAD. Indicators ‘*’ represent HF vs. HF-E1, ‘&’ symbolize HF-E1 vs. HF-E2, and ‘#’ denotes HF-E2 vs. HF comparisons. (**H**) HE staining images displaying myocardial tissue variations across different groups. (**I**) Images of Masson’s trichrome staining of myocardial tissues, indicating differences in fibrosis among groups. (**J**) Quantification of infarct areas based on Masson’s trichrome staining across groups. (**K**) Electron micrographs representing the ultrastructure of cardiomyocytes from various groups. (**L**) Western blot analysis shows the expression of HIF-1α, MCT1, andMPC1 proteins in myocardial tissues, with tubulin as the protein loading control. The one-way ANOVAs followed by Dunnett’s multiple comparison test was used for multi-component comparisons. Mean ± SD. ^*^: *P* < 0.05, ^**^: *P* < 0.01, ^***^: *P* < 0.001, ^&^: *P*<0.05,  ^#^^#^^#^: *P*<0.001. *n* = 6–8

Rats were trained on a treadmill with individual lanes designed for small animals. One week after the ligation surgery (week 1), rats in the HF-E2 group were subjected to a 4-day adaptive exercise (familiarity period) on a zero-inclination treadmill to minimize potential stress; this consisted of the rats running for 3 min at each of four speeds, 8 m/min, 10 m/min, 12 m/min, and 14 m/min on the first day (a total of 12 min), with the speeds and times of this phase (warm-up) remaining unchanged during the subsequent adaptive and formal exercise training; then the rats ran at 16 m/min for 15 min, followed by a gradual increase of 10 min each day until the fourth day when the rats ran at 16 m/min for 45 min; finally, the rats ran at 8 m/min for 3 min, and the speeds and times of this phase (cool down) remained unchanged during the subsequent adaptive and formal exercise training. The rats in the HF-E1 group started adaptive at week 7 with the same adaptive exercise protocol as the rats in the HF-E2 group, followed by 6 weeks of formal exercise training, whereas the rats in the HF-E2 group underwent 12 weeks of formal exercise training. Formal exercise training for rats in the HF-E1 and HF-E2 groups consisted of running at a speed of 16 m/min for 45 min per day (main exercise) in addition to the 12-min warm-up and 3-min cool down described above, at a frequency of 5 days per week, totalling 1 h per day. Rats assigned to HF group (no exercise) were placed on the static treadmill for a matched stage to minimize the impact of the experimental environment on the results during the entire training (Figs. 1A and 2A).

### Echocardiography

Non-invasive cardiac function evaluation was performed by echocardiography in all rats before surgery and at weeks 2,5,10,13 after surgery, respectively (Figs. 1E and 2E). Briefly, an animal anesthesia machine (ventilated 2% isoflurane) was used to anesthetize all rats throughout the surgery. Rats were positioned in the supine position with front paws wide open and ultrasound transmission gel was applied to the precordium. Transthoracic echocardiography was performed using an echocardiographer equipped with a 40-MHz probe, and B-mode images were subsequently obtained in the long and short axis. Cardiac function and structure features such as left ventricular volumes [left ventricular end-diastolic volume (LVEDV) and left ventricular end-systolic volume (LVESV)] and diameters [left ventricular end-diastolic diameter (LVEDD) and left ventricular end-systolic diameter (LVESD)] and fractional shortening (FS) and ejection fraction (EF) were measured and recorded. Left ventricle systolic function was estimated by EF and FS as follows: EF (%) = [(LVEDV − LVESV)/LVEDV] × 100 and FS (%) = [(LVEDD − LVESD)/LVEDD] × 100.

### Measurement of tibial lengths

To calculate heart weight/tibia length (HW/TL), TL in rats anesthetized by a small animal anesthesia machine (1.5% of isoflurane) was measured at the end of exercise training using an animal bone density body composition instrument according to the manufacturer’s protocol.

### Tissue preparation

At the end of the training protocol, 48 h after the last exercise session and after 12 h of fasting, all rats were humanely euthanized with ketamine (50 mg/kg, i.p.) and xylazine (10 mg/kg, i.p.) and body weights (BW) were weighed. All rat hearts were dissected, measured for length and weighed, and myocardial tissues were subsequently collected. Some of the isolated myocardial tissues were fixed with 4% paraformaldehyde/karnovsky fixative solution and others were stored at − 80 °C, these were used for Hematoxylin-eosin (HE) and Masson staining, Electron microscopy analysis and Western blotting, respectively.

### Histological staining and analysis

For staining experiments, the formaldehyde-fixed heart tissue samples were washed with water and underwent gradient dehydration and paraffin embedding. Paraffin-embedded heart samples were transversely cut into 5 μm-thick slices onto slides and subsequently stained with HE and Masson’s trichrome. Each sample was observed under the microscope at six randomly selected visions. The Infarct size presented by Masson staining was quantified using Image J software version 1.8 (National Institutes of Health, Bethesda, MD, USA).

### Electron microscopy analysis

Specific parts of the heart were taken and fixed in 2% glutaraldehyde and 4% paraformaldehyde in sodium acetate buffer at pH 7.3 for 1 h at room temperature and cut into ^~^1-mm^3^ pieces. Samples were washed in phosphate-buffered saline and post-fixed in 2% osmium tetroxide and 1% uranyl acetate for 2 h, rinsed in water, dehydrated in a graded series of ethanol and acetone, and then infiltrated and embedded in Eponate 12 medium. The ultrathin sections were prepared on a Reichert-Jung Ultracut E ultramicrotome (Leica Corporation, Shanghai, China), picked up on copper grids and stained. Images were acquired using a JEM-100CX electron microscope (JEOL Japan Electronics Co., Ltd. Tokyo, Japan) and a 4k digital camera (Gatan Orius 4k X).

### Cell culture and hypoxia cell model

H9c2 cells (rat embryonic cardiomyoblast-derived H9c2 cardiomyocytes) were purchased from the Cell Bank of the Chinese Academy of Sciences (Shanghai, China). H9c2 cells were maintained at 37 °C in a 5% CO_2_ incubator containing complete culture medium (DMEM and 10% fetal bovine serum supplemented with 1% of a penicillin/streptomycin solution) and passaged at approximately 90% confluency. Cell morphologic changes were observed under an inverted fluorescence microscope (CKX53-LP, OLYMPUS CK31MIF-BGU-LED). To establish the hypoxia model, H9c2 cells were cultured in hypoxia incubator with 1% O_2_, 5% CO_2_ and 94% N_2_ for 24 h and 48 h. H9c2 cells in the normoxia group were cultured at 37℃ in a normoxic incubator containing 21% O_2_ and 5% CO_2_ and served as the control. Hypoxic H9c2 cells were treated with the HIF-1α activator desferrioxamine mesylate (DFO, 20µM, dissolved in dimethyl sulfoxide, MCE, HY-B0988) (Dziegala et al. 2016) and the HIF-1α inhibitor KC7F2 (20µM, dissolved in dimethyl sulfoxide, MCE, HY-18,777) (Li et al. 2022) for 48 h.

### Cell transfection

Three target shRNA plasmids (HIF-1α-RNAi-Easy-shRNA, MCT1-RNAi-Easy-shRNA and MPC1-RNAi-Easy-shRNA) and a negative control (Control-RNAi-shRNA) were developed and synthesized by the manufacturer (Ji Kai Gene Technology Co., Ltd, Shanghai, China), and we subsequently transfected them into hypoxic H9c2 cells using Lipo3000 transfection reagent (GK20006-25, GLPBIO) according to the manufacturer’s instructions. Our transfected plasmid contained the green fluorescent protein gene, and strong fluorescence intensity was observed in hypoxic H9c2 cells under fluorescence microscope, which indicated the high efficiency of cell transfection (Fig. 4A). Briefly, H9c2 cells (5 × 10^5^) were grown in 6-well plates for 24 h. Lipo3000 was diluted using serum-free medium and allowed to stand for 5 min at room temperature. The diluted shRNA was mixed with medium containing Lipo3000 transfection reagent and incubated at room temperature for 15 min, and then the DNA-lipid complex was added into the cells. The transfected cells were grown in hypoxia incubator for 24 h and tested for gene silencing level and expression of each group of indicators.

### Cell viability test

The effect of hypoxia on the proliferation of H9c2 cells was detected using the CCK8 Cell Proliferation Kit (Beyotime, Biological Co., Ltd, Beijing, China). H9c2 cells (100 µL) were grown at 2 × 10^4^ cells/mL in 96-well plates overnight. After incubation for 24 h, the cells were treated with hypoxia for 24 and 48 h. After treatment, 100 µL of media enriched with 10% CCK-8 solution was added to the cells through media exchange modes and incubated for 1 h. Absorbance was measured at 450 nm using a Multi-mode Microplate Reader (Molecular Devices, San Jose, CA, USA). Each experiment was performed in sextuplicate, and cell survival rates were expressed as a percentage of the control.

### Comparative analysis of ATP

The ATP level analyzed with ATP assay kit (Beyotime, Biological Co., Ltd, Beijing, China). The experimental procedure was consistent with the instruction of the manufacturer. The absorbance readings for the determination of ATP were carried out at 560 nm via the detection of absorbance of a multi-well plate in a Multi-mode Microplate reader (Finalytek, USA). It was able to use this approach to quantify ATP levels in different groups of cells.

### Assessment of cell apoptosis

After hypoxia treatment of H9c2 cells, an Annexin V-FITC/PI apoptosis kit (Keygentec, Nanjing, China) was used for cell staining and flow cytometry following the manufacturer’s instructions. Briefly, cells from each group was washed twice with PBS and 2 × 10^5^ cells were reconstituted with 500µL of binding buffer. Subsequently, 5µL of Annexin V-FITC was immediately added and mixed well. Then another 10µL of PI was added to the cells and incubated at room temperature for 5 minutes in the dark, ready for apoptosis analysis on an Accuri C6 flow cytometer (BD Biosciences, San Jose, CA, USA).

### Immunofluorescence analysis

Cell immunofluorescence was performed by fixing cells with 4% tissue cell fixative (Solarbio, Beijing, China) for 30 min at room temperature, rinsing once with PBS, and then permeabilising with 0.1% Triton X-100 (BioFroxx) for 15 min. Subsequently, the cells were non-specifically blocked with 1% BSA (BioFroxx) for 1 h at room temperature, followed by anti-HIF-1α (20960-1-AP, 1:200, Proteintech), anti-MCT1 (A3013, 1:100, ABclonal) and anti-MPC1 (A20195, 1:100, ABclonal) were incubated overnight at 4 °C. Next, cells were reacted with Alexa Fluor® 594-conjugated goat anti-rabbit IgG secondary antibody (ZF-0516, ZSZSGBBIO) and FITC-labeled goat anti-rabbit IgG secondary antibody (ZF-0311, ZSZSGBBIO) at 1:50 for 30 min at 37 °C protected from light, respectively, followed by reaction with DAPI solution (C0065, Solarbio) for 5 min at room temperature. Finally, imaging was performed immediately on an inverted fluorescence microscope (CKX53-LP).

### Western blotting

Briefly, protein extracts obtained from isolated myocardial tissue or cultured cardiomyocytes were homogenized in RIPA lysis buffer (Solarbio Life Sciences, R0020, China) containing phenylmethylsulfonyl fluoride (PMSF). The homogenate was centrifuged at 10,000× g for 10 min at 4 °C and supernatant collected, and the total protein concentration was subsequently determined using the BCA Assay Kit (Solarbio Life Sciences, PC0020, China). 5X sample loading buffer was added proportionally to the protein-containing supernatant and boiled (100 °C, 10 min) to obtain protein samples. Protein samples (20 µg) were loaded with standard marker proteins at room temperature and electrophoretically separated (250 mA, 100 min) on polyacrylamide gels (15% Sure-PAGE™, Genscript, China), and then the proteins were transferred onto polyvinylidene fluoride membranes (PVDF, Millipore, USA). Next, membranes were blocked for 2 h at room temperature with 5% defatted milk in TBST (0.1% Tween 20 in TBST) and incubated overnight at 4 °C with the following primary antibodies: HIF-1α (ab179483, 1:1000, Abcam, USA), MCT1 (A3013, 1:1000, ABclonal, China), MPC1 (A20195, 1:1000, ABclonal, China), β-tubulin (380,628, 1:5000, Zen BioScience, China). The membrane was repeatedly rinsed with TBST buffer three times for 10 min each, and incubated with horseradish peroxidase (HRP) conjugated IgG antibody-Goat Anti-Rabbit IgG H&L/HRP (bs-40295G-HRP, 1:10000, Bioss, China) for 1 h at room temperature. After incubation with the secondary antibody, the membrane was repeatedly rinsed three times for 10 min each with TBST buffer and treated with enhanced chemiluminescence reagent (ECL, WBKLS0500, Millipore, USA). Subsequently, protein bands were visualized via chemiluminescent detection in a gel image processing system (Amersham Imager 680, CTL, USA), and quantified by densitometry using Image J analysis software version 1.8. Targeted bands were normalized to the relative expression of cardiac β-tubulin.

### Statistical analysis

The results are reported as the Mean ± SD. All data were statistically analyzed and graphed using SPSS software (version 22.0) and GraphPad Prism (version 8.0). The unpaired two-tailed Student t-test was used for comparison between two groups, and one-way ANOVAs followed by Dunnett’s multiple comparison test was used for multi-component comparisons. All experiments were conducted at least three biologically independent replicates. Statistical significance was accepted at ^*^p < 0.05, ^**^p < 0.01, ^***^p < 0.001.

## Results

### Pathological remodelling occurs in the failing heart

To investigate the effects of exercise on the pathophysiology of failing hearts, we first measured cardiac morphology and function in a rat model of MI. Cardiac morphological analysis showed typical pathological pallor and left ventricular (LV) thinning in HF group compared to the SHAM group (Fig. 1B). Heart hypertrophy indices (HWI, HW/BW) and HW/TL ratios were significantly higher in the HF group than in the SHAM group 13 weeks post-surgery, with abnormal cardiac hypertrophy (Fig. 1C and D). Echocardiography (Fig. 1E) demonstrated cardiac dysfunction with LV dilatation (LV internal diameters) in HF rats. FS (Fig. 1F) and EF (Fig. 1G) of rats in HF group markedly reduced relative to that of SHAM group, thereby indicating cardiac dysfunction in the HF group. HE staining highlighted myocardial fiber deformation (black arrows), hypertrophic cardiomyocytes (yellow arrows), and telangiectasia with hyperemia (red arrows) in the HF group compared with the SHAM group (Fig. 1H). Masson staining manifested deeper myocardial fibrosis (black arrows) and increased infarct size (Fig. 1I and J) and severe ultrastructural damage in myocardial fibers by electron microscopy in the HF group compared with the SHAM group (Fig. 1K). Interestingly, our data illustrated that the levels of HIF-1α (*p* < 0.01), MPC1 (*p* < 0.001) and MCT1 (*p* < 0.001) protein expression were significantly higher in HF group of rats than in SHAM group (Fig. 1L). Together, at this stage, post-MI animals presented a range of pathologic remodelling imagery and eventually developed HF with an accompanying reduction in cardiac metabolic capacity.

### Treadmill exercise training improves MI-induced cardiac pathological remodelling

Next, we determined whether exercise could improve pathological remodelling in HF. Significant benefits in morphology, structure and cardiac function were observed in the HF-Exercise group at the end of the protocol. Overall, exercise significantly alleviated cardiac pallor in rats with MI, suppressed LV thinning, attenuated MH, prevented LV dilatation, enhanced LV contractility and ejection function (FS and EF notably increased), and ultimately delayed the course of HF (Fig. 2B-G). Similarly, exercise training ameliorated the organic changes in the infarcted myocardium, mainly in the form of inhibition of pathological hypertrophy of cardiomyocytes, enlargement of the myocardial space, reduction of the degree of myocardial fibrosis and infarct area, and restoration of the ultrastructure of myocardial fibres (Fig. 2H-K). Our results indicated that exercise training markedly improved cardiac function and re-established pathological remodelling progression in rats with MI. As noted above, these findings reinforce previous research data (Peixoto et al. 2015) that better pathological characteristics from exercise training are an important outcome in improving cardiovascular disease, suggesting that exercise training intervention was very efficient. One possible explanation for the benefits of exercise in HF could be attributed to altered metabolic processes and responses to hypoxia. Therefore, we then characterized the effects of exercise on HIF-1α, MCTI and MPC1 in rats with MI. The protein expressions of cardiac HIF-1α were detected using Western blot for all groups. Of interest, compared with the HF group, exercise training significantly upregulated the expression of HIF-1α, MCT and MPC1 proteins (Fig. 2L). We found that exercise training reversed the LAD-induced down-regulation of HIF-1α expression levels, indicating that the improvement in HF with prolonged exercise may be due to increased expression of key metabolic proteins.

### Hypoxia triggers H9c2 cell injury in vitro

**Fig. 3:**
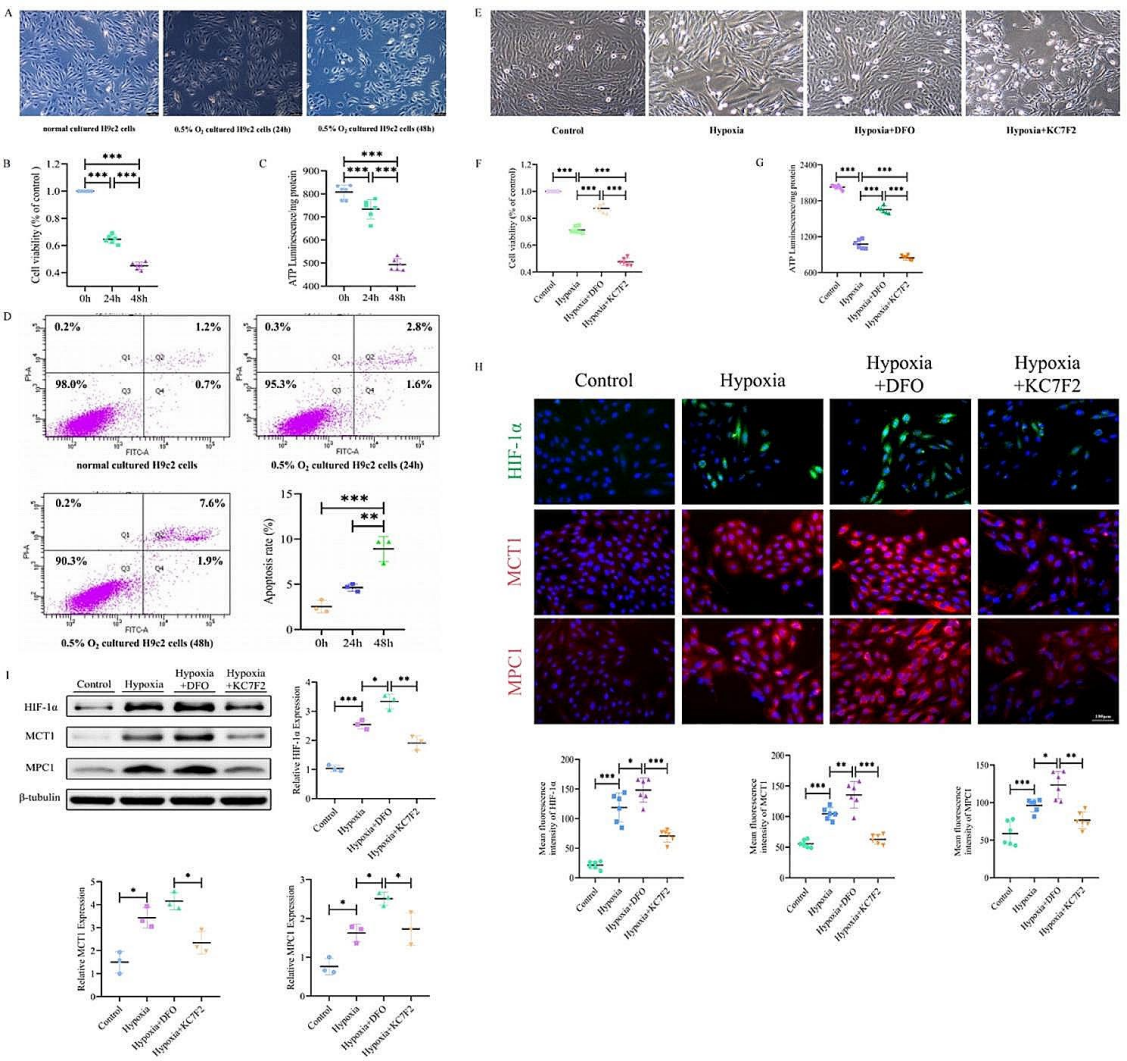
HIF-1α pharmacological efficacy alters the hypoxic injury model in H9c2 cells. (**A**) Visual representation of hypoxia-induced morphological changes in H9c2 cells. (**B**) Graph showing the impact of hypoxia on H9c2 cell viability across different time points. (**C**) Graph illustrating the effect of hypoxia on intracellular ATP levels in H9c2 cells over time. (**D**) Graph representing the influence of hypoxia on apoptotic levels in H9c2cells over time. (**E**) Visual representation of DFO and KC7F2 affecting morphological alterations in H9c2 cells. (**F**) Graph illustrating the effects of DFO and KC7F2 on H9c2 cell viability under hypoxic conditions. (**G**) Graph showing the effects of DFO and KC7F2 on intracellular ATP levels in H9c2 cells under hypoxic conditions. (**H**) Immunofluorescence analysis reveals the effects of DFO and KC7F2 on the expression levels of HIF-1α, MCT1 and MPC1 in hypoxic H9C2 cells. (**I**) Western blot analysis depicting the effects of DFO and KC7F2 on the expression levels of HIF-1α, MCT1 and MPC1 protein expression in hypoxic H9C2 cells. The one-way ANOVAs followed by Dunnett’s multiple comparison test was used for multi-component comparisons. Data represent Mean ± SD. Of at least three independent experiments (*n* = 3 per group).^*^: *P* < 0.05, ^**^: *P* < 0.01, ^***^: *P* < 0.001

The hypoxia protocol we implemented was intended to mimic the oxygen-deprived environment of ischemic failing heart, suggesting that hypoxia after MI can disrupt cardiomyocyte metabolism and may act as a contributing factor to alterations in the pyruvate-lactate axis. To explore this, we next used differentiated H9c2 cells to better elucidate the impact of hypoxia on cell morphology, cell viability, ATP levels, and apoptosis rates. We found that compared to the normoxia-treated group (cells arranged in a spindle shape and well-grown), sustained hypoxia treatment for 48 h markedly enhanced H9c2 cell death, decreased cell survival (fewer cells), and resulted in abnormal morphological changes (obviously rounded cells) (Fig. 3A). With the prolonged duration of hypoxia (24 h and 48 h), both H9c2 cell viability and ATP levels were significantly decreased compared to the normoxic group (Fig. 3B and C). Additionally, the early and late apoptosis rates of H9c2 cells at 48 h of hypoxia were significantly higher than those of the normoxia-treated group (Fig. 3D). Therefore, these effects of hypoxia on cells are time-dependent and are accompanied by structural damage, reduced numbers, and decreased ATP levels.

### Pharmacological activation/inhibition and knockdown of HIF-1α alters MCT1 and MPC1 expression

Our study further explored the beneficial utility of exercise in the failing heart, focusing on the effects of pharmacological activation/inhibition of HIF-1α on MCT1 and MPC1 in hypoxic cardiomyocytes.We treated hypoxic H9c2 cells with KC7F2 (a HIF-1α inhibitor) and DFO (a widely used HIF-1α activator to simulate the key effects of exercise) respectively and served as an experimental group (hypoxia + KC7F2, hypoxia + DFO), and used normal and hypoxic cells as two control groups (control and hypoxia). We observed that pharmacological activation of HIF-1α reduced the cellular damage caused by hypoxia (increased cell number and improved morphology), restored and significantly increased cell viability and ATP levels (Fig. 3E-G); interestingly, overexpression of HIF-1α led to elevated levels of HIF-1α, MCT1, and MPC1 expression under the same conditions (Fig. 3H and I). Conversely, pharmacological inhibition of HIF-1α in hypoxia (hypoxia + KC7F2) reversed these phenomena compared to the hypoxia + DFO group, suggesting that the negative effects of KC7F2 could be corrected by increasing HIF-1α levels. The above data demonstrate that HIF-1α reduction exacerbates the damage and energy deficiency of cardiomyocytes under hypoxic conditions; whereas HIF-1α overexpression triggers expression of MCT1 and MPC1 proteins and enhances energy production in cardiomyocytes, suggesting its pivotal role in the repair of hypoxic cardiomyocytes.

**Fig. 4:**
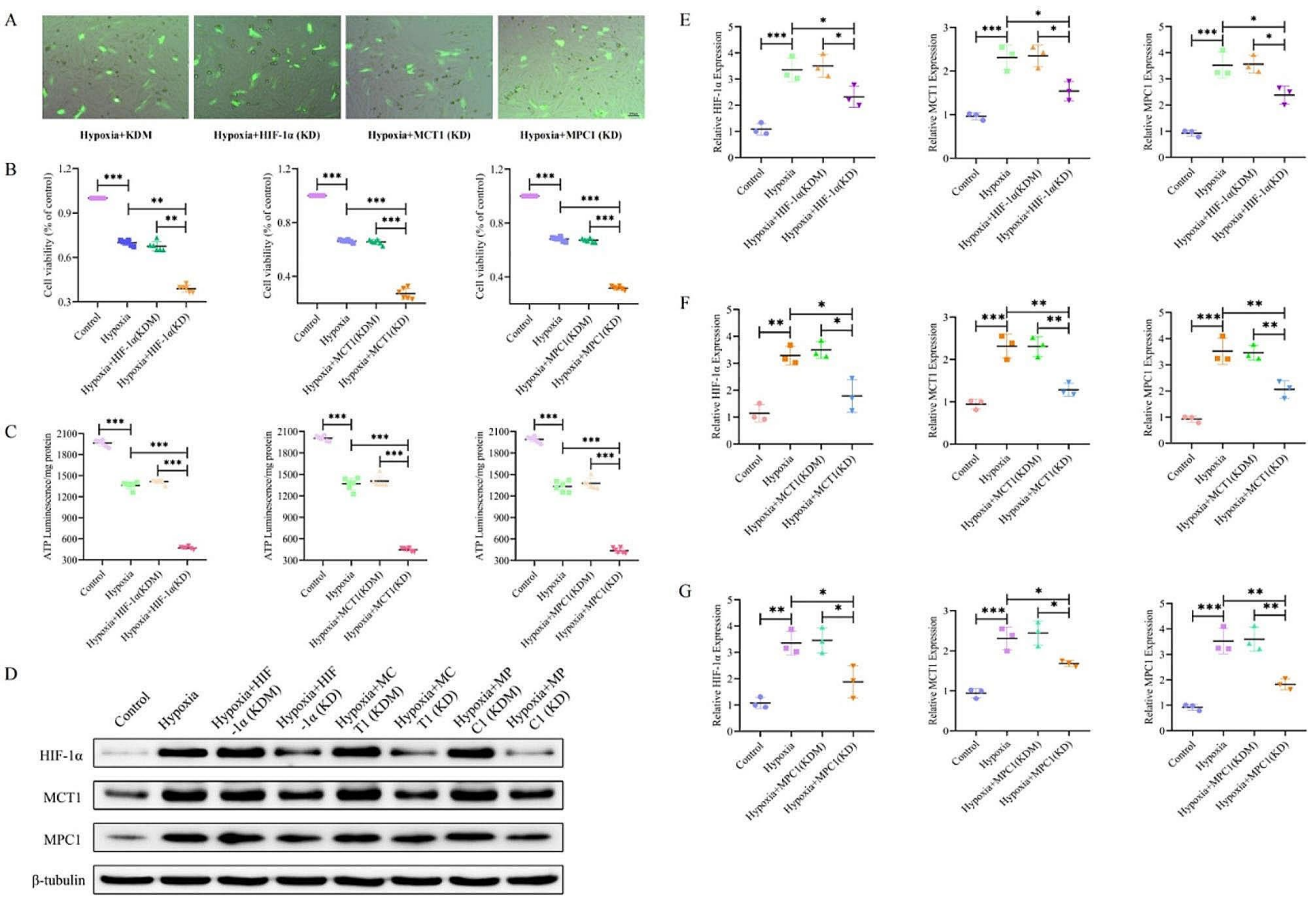
Effects of HIF-1α, MCT1 and MPC1 knockdown on hypoxic H9c2 cells. (**A**) Visual representation of fluorescence after shRNA transfection targeting HIF-1α, MCT1 and MPC1 in hypoxic H9c2 cells. (**B**) Graph depicting the impact of HIF-1α, MCT1 and MPC1 knockdown on H9c2 cell viability under hypoxic conditions. (**C**) Graph showing the effects of HIF-1α, MCT1 and MPC1 knockdown on intracellular ATP levels in H9c2 cells under hypoxic conditions. (**D-G**) Western blot analysis demonstrating the effect of HIF-1α, MCT1 and MPC1 knockdown on the expression levels of HIF-1α, MCT1 and MPC1 proteins in hypoxic H9C2 cells. The one-way ANOVAs followed by Dunnett’s multiple comparison test was used for multi-component comparisons. Data represent Mean ± SD. Of at least three independent experiments (*n* = 3 per group).^*^: *P* < 0.05, ^**^: *P* < 0.01, ^***^: *P* < 0.001

In order to shed new light on mechanisms recruited by HIF-1α changes on MCT1 and MPC1, we performed knockdown experiments for each of the three target genes in hypoxic H9c2 cells. For that, hypoxic H9c2 cells were transfected with shRNA targeting the HIF-1α, MCT1 and MPC1 gene (Fig. 4A) to knock down HIF-1α, MCT1 and MPC1 expression (KD, knock down group); meanwhile, negative control shRNA served as a comparator (KDM, knock down mock group). The CCK-8 assay revealed that the cell viability was apparently lower in the HIF-1α, MCT1 and MPC1 knockdown group compared to KDM (Fig. 4B). ATP levels were reduced upon HIF-1α, MCT1 and MPC1 gene knockdown in hypoxic cardiomyocytes receiving shRNA compared to KDM (Fig. 4C). The results of western blot experiments showed that knockdown of the HIF-1α gene significantly reduced HIF-1α protein expression compared with Hypoxia + HIF-1α (KDM). Interestingly, HIF-1α targeted silencing also leads to decreased MCT1 and MPC1 protein expression in hypoxic cardiomyocytes compared with Hypoxia + HIF-1α (KDM) (Fig. 4D and E); and knockdown of MCTI and MPC1 leads to results consistent with this (Fig. 4F and G). Collectively, these available results replicate in vitro the beneficial effects of exercise training on the failing heart and supports the hypothesis that the improvement in HF via MCT1 and MPC1 is mediated through exercise-regulated HIF-1α protein enhancement.

## Discussion

Here, we provide compelling evidence that (a) exercise training attenuates myocardial injury and dysfunction in a rat model of MI. (b) The cardioprotective effect of exercise training was associated with enhanced expression of HIF-1α and up-regulated expression of MCT1 and MPC1, suggesting that changes in the levels of key metabolic proteins post-MI may be a positive factor in the improvement of cardiac dysfunction. (c) In H9c2 cardiomyocytes, the upregulation of MCT1 and MPC1 via HIF-1α overexpression enhances cellular metabolism, potentially elucidating the molecular mechanisms underlying exercise-induced delay in HF progression.

HF involves post MI adaptations and remodelling of the myocardium at the structural, cellular, humoral and molecular levels, and is characterised by a reduced capacity of the heart to pump or fill with blood, typically preceded by pathological MH manifested by an abnormal enlargement of cardiomyocytes and accompanied by the dysregulation of key effector molecules (Tham et al. 2015), and ultimately by a decline in cardiac function, which are evoked to maintain viable cardiac output and systemic circulation. Preceding studies demonstrated that MI leads to early necrosis/apoptosis of cardiomyocytes and LV dysfunction (Unsöld et al. 2014), followed by compensatory hypertrophy and cardiomyocyte thickening in survived myocardium to compensate for cardiac disease (Nakamura and Sadoshima 2018); furthermore, cardiac injury disrupts the balance between fibroblasts and cardiomyocytes, generating a state favoring inflammation and fibrosis (Kyselovič and Leddy 2017), which eventually leads to pathological remodeling and devastating outcomes of HF. Using an in vivo model of post MI-induced HF, we provided evidence that failing hearts exhibited typical pallor, pathological hypertrophy of cardiomyocytes, abnormal MH and fibrosis, and increased infarct size with concomitant myocardial ultrastructural damage. These changes were followed by deeper infarction, cardiac dysfunction with reduced bioenergetic efficiency. Our present results conceptually support the notion of a series of pathological features enumerated in post-MI hearts as reported above. Among many investigated strategies, exercise training serves as a natural, non-pharmacological cardioprotective stimulus that can induce prolonged or sustained cardioprotective state (Alánová et al. 2017), and it has also been recommended for improving cardiac function and quality of life in post-MI patients (Fletcher et al. 2013). The results of our current rodent study essentially confirm that exercise training post-MI reverses symptoms caused by pathological hypertrophy, improves morbid cardiac remodelling and systolic disorder, and contributes to normalization of myocardial metabolism and cardiac function, which is in line with several previous studies (Johnson et al. 2015; Jia et al. 2019). It is notable that our abovementioned in vivo heart data were further validated in cultured cardiac cells in vitro. In H9c2 cells, we observed that prolonged hypoxia triggered cellular structural damage, reduced cell viability and ATP levels, and induced cell injury and apoptosis, similar to what we found in another study (Liang et al. 2020).

It has been established that cellular responses to exercise is largely achieved through activation of HIF-1 (Tekin et al. 2010), which functions as a key oxygen sensor that senses and coordinates the cellular response to hypoxia and protects the cell by controlling oxygen delivery and utilization. One study demonstrated that 8 weeks of exercise training inhibited pathological hypertrophy of the hearts and preserved cardiac microvessel density in mice through successive HIF-1α/VEGF (vascular endothelial growth factor) up-regulation in endothelial cells during continued pressure overload (Tian et al. 2020). Another study (animal and cellular experiments) found that HIF-1α, expressed upregulated during 4 weeks of exercise training, promotes myocardial angiogenesis through activation of the Phosphatidylinositol 3-kinase (PI3K)/ Protein kinase B (Akt)/ endothelial nitric oxide synthase (eNOS) signalling pathway and subsequently improves cardiac function post-MI in rats (Song et al. 2020). Again, there are corresponding findings in our experiments. These results are explained, at least in part, by the positive effect of exercise-upregulated HIF-1α on the improvement of cardiac function in rats post-MI. Interestingly, our study found that long-term exercise upregulation of HIF-1α protein expression in heart failure rats was accompanied by increased myocardial MCT1 and MPC1 expression. Furthermore, a study has demonstrated that 6 weeks of treadmill exercise up-regulated myocardial MCT1 expression in rats with MI, enhanced myocardial glycolytic metabolism potential, and exerted a beneficial role in the remodelling process of the failing heart (Hashimoto et al. 2004), this finding reinforce our in vivo model.

**Fig. 5:**
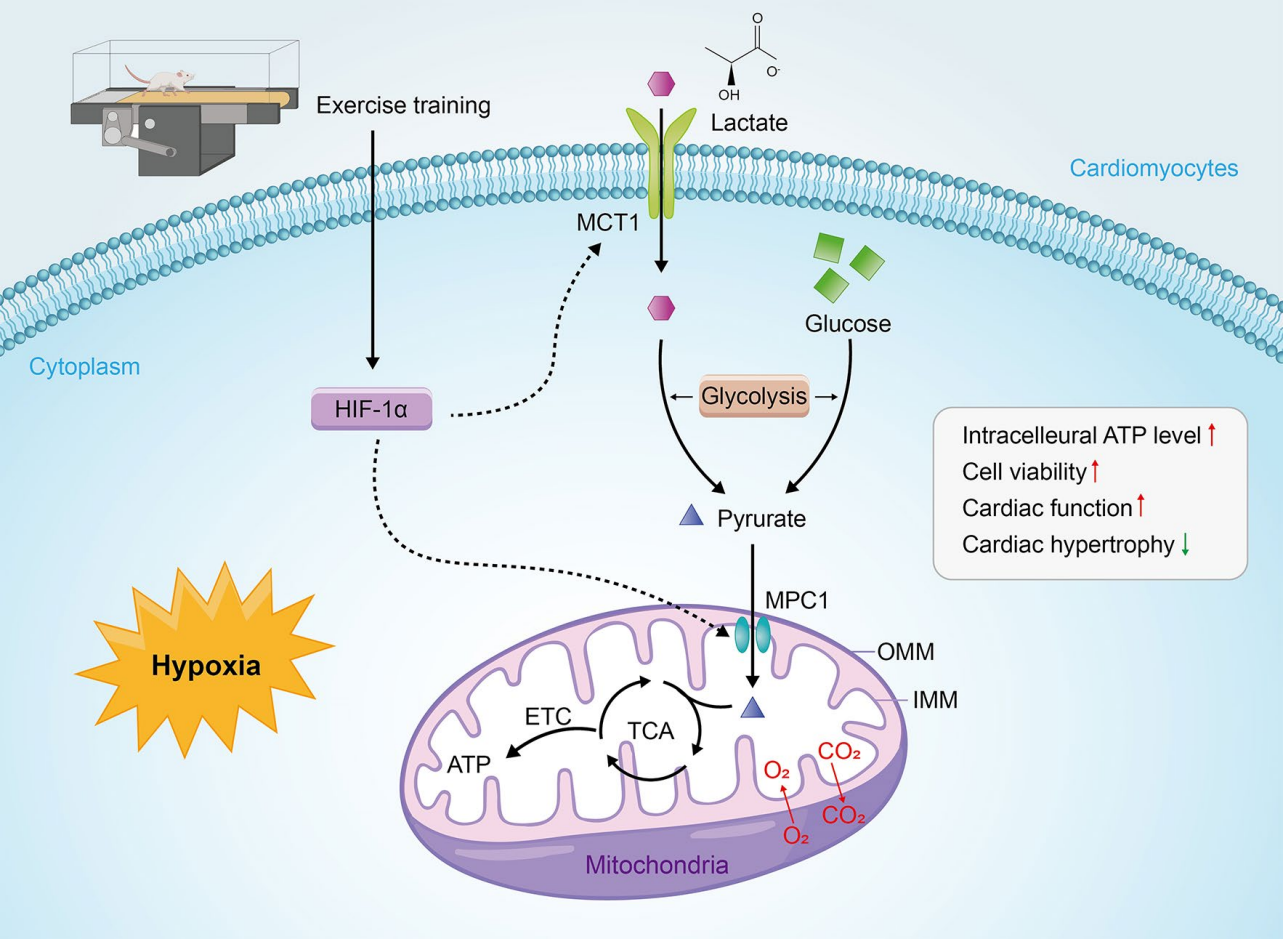
Possible mechanisms by which exercise regulates the failing heart post-MI via HIF-1α. Under hypoxic conditions, exercise increases HIF-1α expression in the failing heart and up-regulates cardiomyocyte MCT1 and MPC1 expression in a HIF-1α-dependent manner to enhance glycolytic processes (increased capacity of cardiomyocytes to transport and uptake lactate), elevate ATP levels and cell survival, and ultimately improve cardiac function. ETC, electron transport chain; IMM, inner membrane of the mitochondria; OMM, outer membrane of mitochondria

A feature of progressively deteriorating myocardial systolic function is inadequate in energy supply due to diminished mitochondrial oxidative phosphorylation rates, causing a decrease in ATP synthesis and phosphocreatine storage. By using a specific HIF-1α inhibitor (KC7F2), we found significant down-regulation of MCT1 and MPC1 protein expression in the hypoxia model, accompanied by a reduction in cell viability and ATP levels; similarly, we silenced HIF-1α using siRNA, which resulted in results consistent with HIF-1α inhibition, with both attenuating the protective effects of HIF-1α. Another interesting finding of the present study is that DFO (HIF-1α activator and exercise memetic) induced a significant up-regulation of MCT1 and MPC1 protein expression in hypoxic H9c2 cells, which was accompanied by restoration and elevation of cell viability and ATP levels, suggesting the multifactorial genesis of cytoprotection by HIF-1α overexpression or presumably exercise. In an in vitro cardiomyocyte model, genetic depletion and loss of activity as well as constitutive knockout of MPC1 in adult mice were sufficient to induce myocardial pathologic hypertrophy, chronic HF, and death; conversely, inducible cardiomyocyte overexpression of MPC1 mitigated drug (tamoxifen)-induced cardiac hypertrophy and failure, increased TCA intermediates and mouse survival, and improved cardiac function  (Cluntun et al. 2021; Fernandez-Caggiano et al. 2020). Therefore, we knocked down the MPC1 gene in hypoxic H9c2 cells and found that it downregulated HIF-1α and MCT1 expression as well as ATP levels. These findings from previous studies provide a plausible explanation for the results we observed in hypoxic cardiomyocytes after HIF-1α overexpression (elevated MPC1 expression). Consistent with these theories, we hypothesized that the reduced MPC1 expression we observed in HF rat cardiomyocytes could underlie the switch to anabolism that culminates in maladaptive hypertrophic growth and failure, which is reversed by elevated MPC1. Based on the aboveinforce our in vivo observations and the growing body of literature on exercise to ameliorate HF, we inferred that exercise enhances the potential capacity of the myocardium to uptake and deliver lactate and oxidize it as an energy substrate, strengthening glycolytic processes, and ultimately compensating the ischemic regions in the heart by increasing myocardial HIF-1α expression in rats with MI possibly up-regulating MCT1 and MPC1 expression in a HIF-1α-dependent manner (Fig. 5).

## Conclusion

Taken together, this study clarifies the process by which exercise training ameliorates MI-initiated cardiac remodelling and systolic ailment, as well as the crucial role of HIF-1α-dependent lasting preservation of the up-regulation of MCT1 and MPC1 in response to exercise training. We provide evidence that exercise training primarily stimulates high myocardial HIF-1α and MCT1 and MPC1 expression, increases cardiomyocyte survival and bioenergetic efficiency, promotes cardiometabolic adaptation, and exerts a protective effect by attenuating pathological features and ameliorating cardiac dysfunction in a HF model. This current finding may provide preliminary theoretical support and insights for studying the regulatory mechanisms by which exercise ameliorates HF, and further studies are required to validate this hypothesis. We hope that our understanding lay the foundation for further detailed studies.

### Electronic supplementary material

Below is the link to the electronic supplementary material.

Supplementary Material 1

## Data Availability

Data and materials are available on request.

## Abbreviations

Akt: Protein kinase B
ATP: Adenosine triphosphate
BW: Body weights
DFO: Desferrioxamine mesylate
ECL: Enhanced chemiluminescence reagent
EF: Ejection fraction
eNOS: Endothelial nitric oxide synthase
FS: Fractional shortening
HE: Hematoxylin-eosin
HF: Heart failure
HIF-1: Hypoxia-inducible factor-1
HIF-1α: Hypoxia-inducible factor-1α
HRP: Horseradish peroxidase
HW: Heart weight
HWI: Heart hypertrophy index
LAD: Permanent ligation of the left anterior descending coronary artery
LV: Left ventricular
LVEDD: Left ventricular end-diastolic diameter
LVEDV: Left ventricular end-diastolic volume
LVESD: Left ventricular end-systolic diameter
LVESV: Left ventricular end-systolic volume
MCT1: Monocarboxylic acid transporter protein 1
MH: Myocardial hypertrophy
MI: Myocardial infarction
MPC1: Mitochondrial pyruvate carrier 1
PI3K: Phosphatidylinositol 3-kinase
PMSF: Phenylmethylsulfonyl fluoride
PVDF: Polyvinylidene fluoride
TCA: Tricarboxylic acid cycle
TL: Tibia length
VEGF: Vascular endothelial growth factor
