# Supplementary material for: Aerobic exercise-induced HIF-1α upregulation in heart failure: exploring potential impacts on MCT1 and MPC1 regulation
Source: Mol Med. 2024 Jun 12;30:83. doi: 10.1186/s10020-024-00854-3 (PMC11167843; doi:10.1186/s10020-024-00854-3)

Figure 1 Pathological remodeling in failing hearts post-myocardial infarction

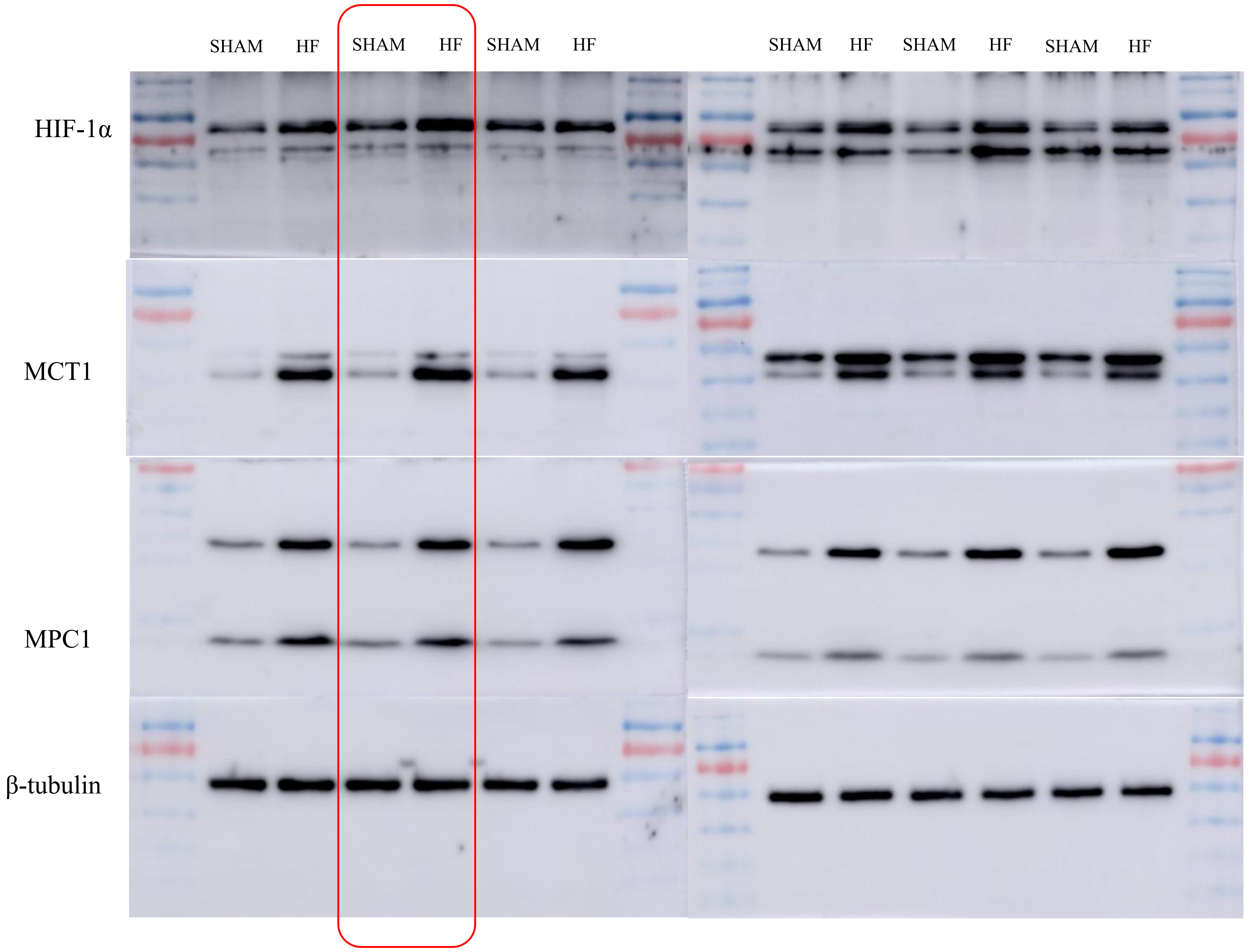

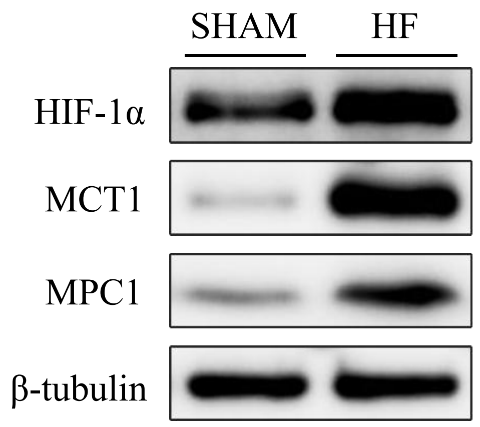

Figure 2 Treadmill exercise training improves pathologic features of the failing heart.

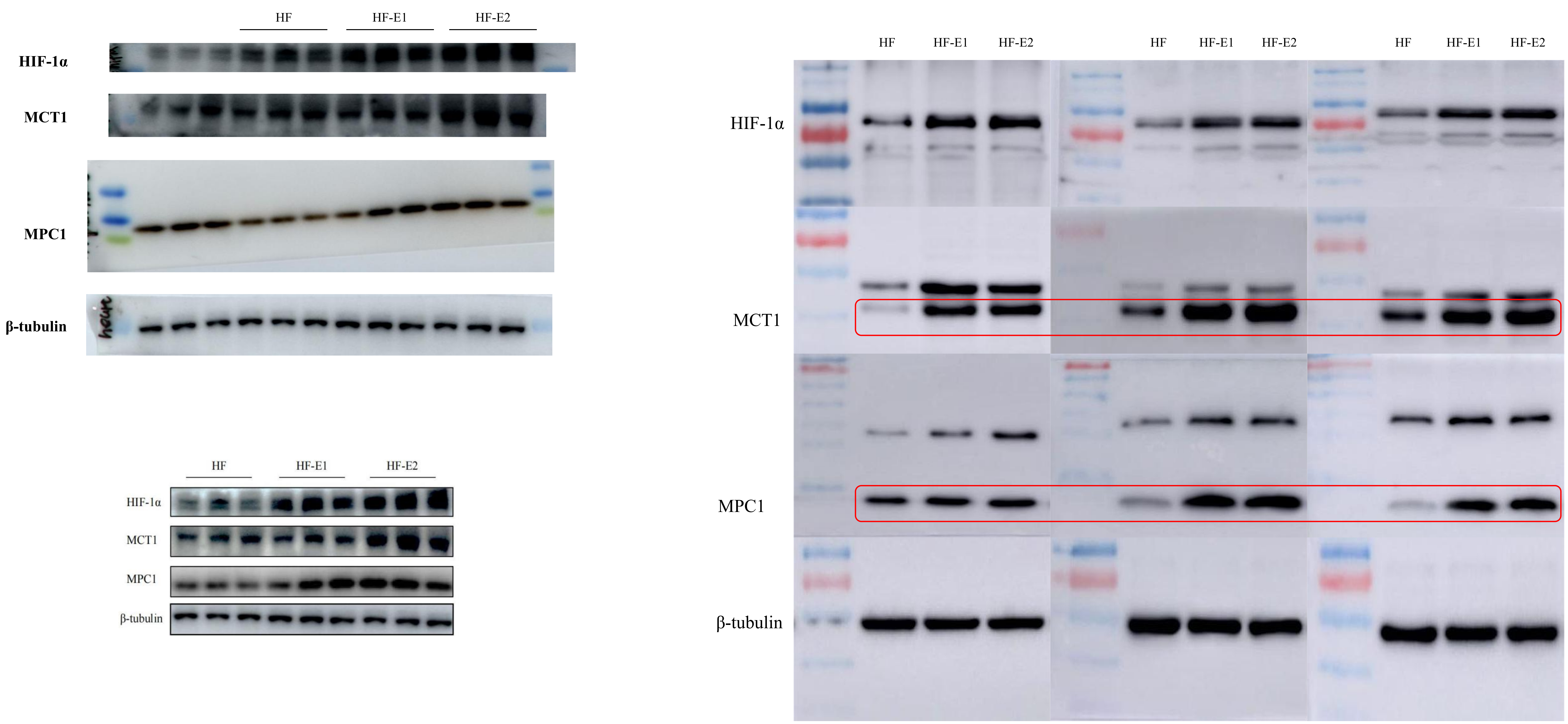

Figure 3 HIF-1α pharmacological activation (KC7F2) and inhibition (DFO) alters the hypoxic injury model of H9c2 cells

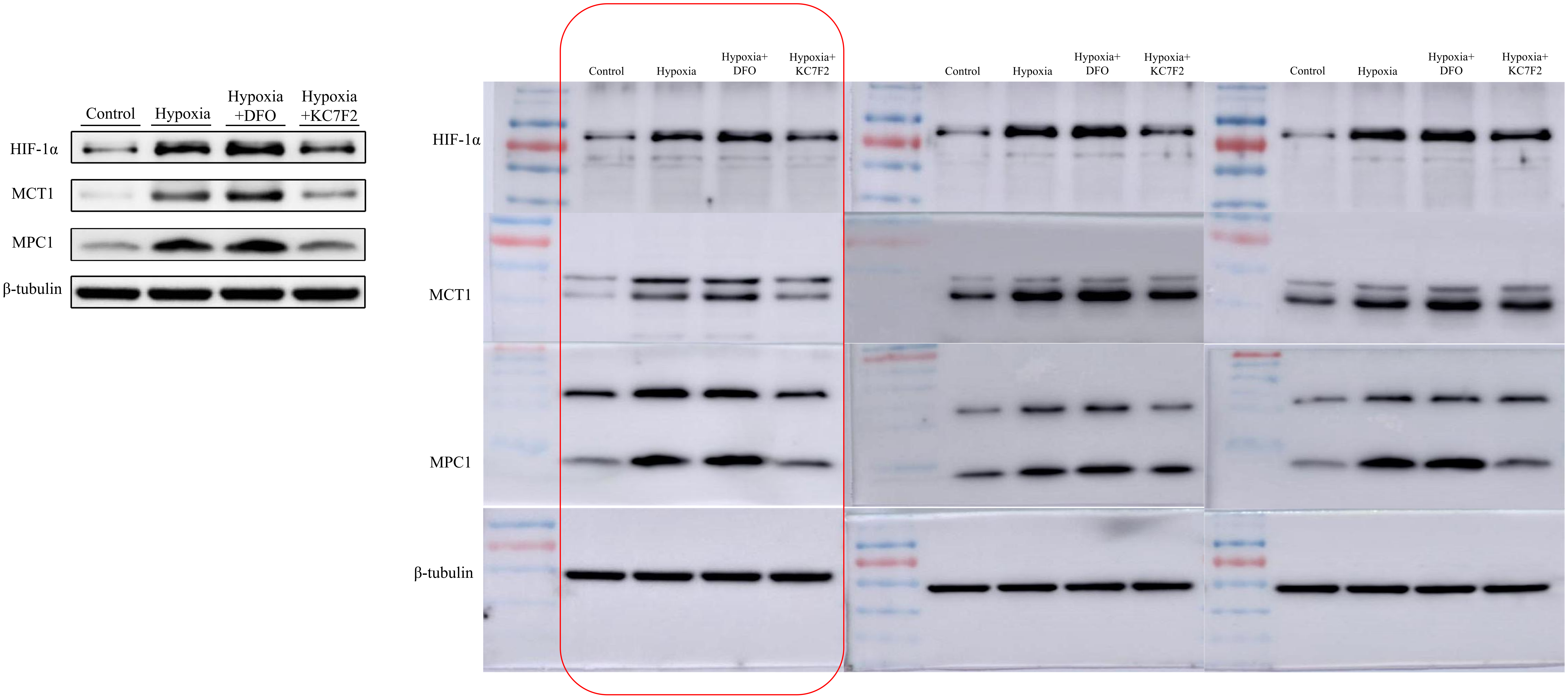

Figure 4 Effects of HIF-1α, MCT1 and MPC1 knockdown on hypoxic H9c2 cells.

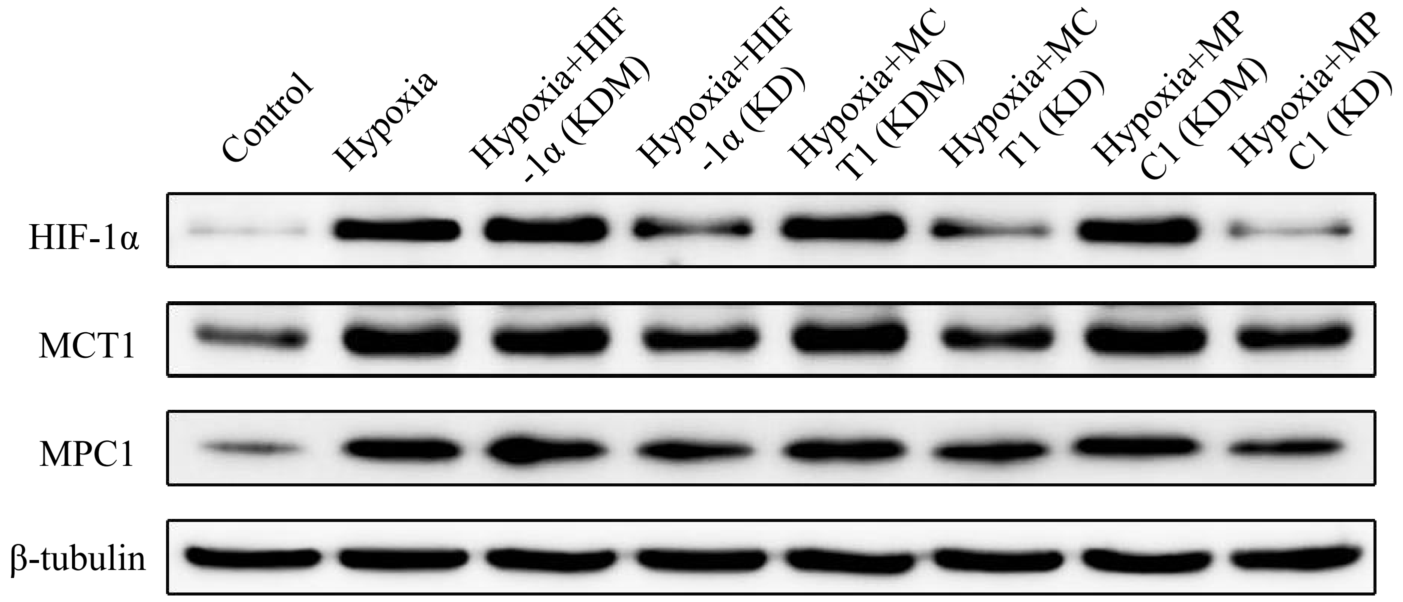

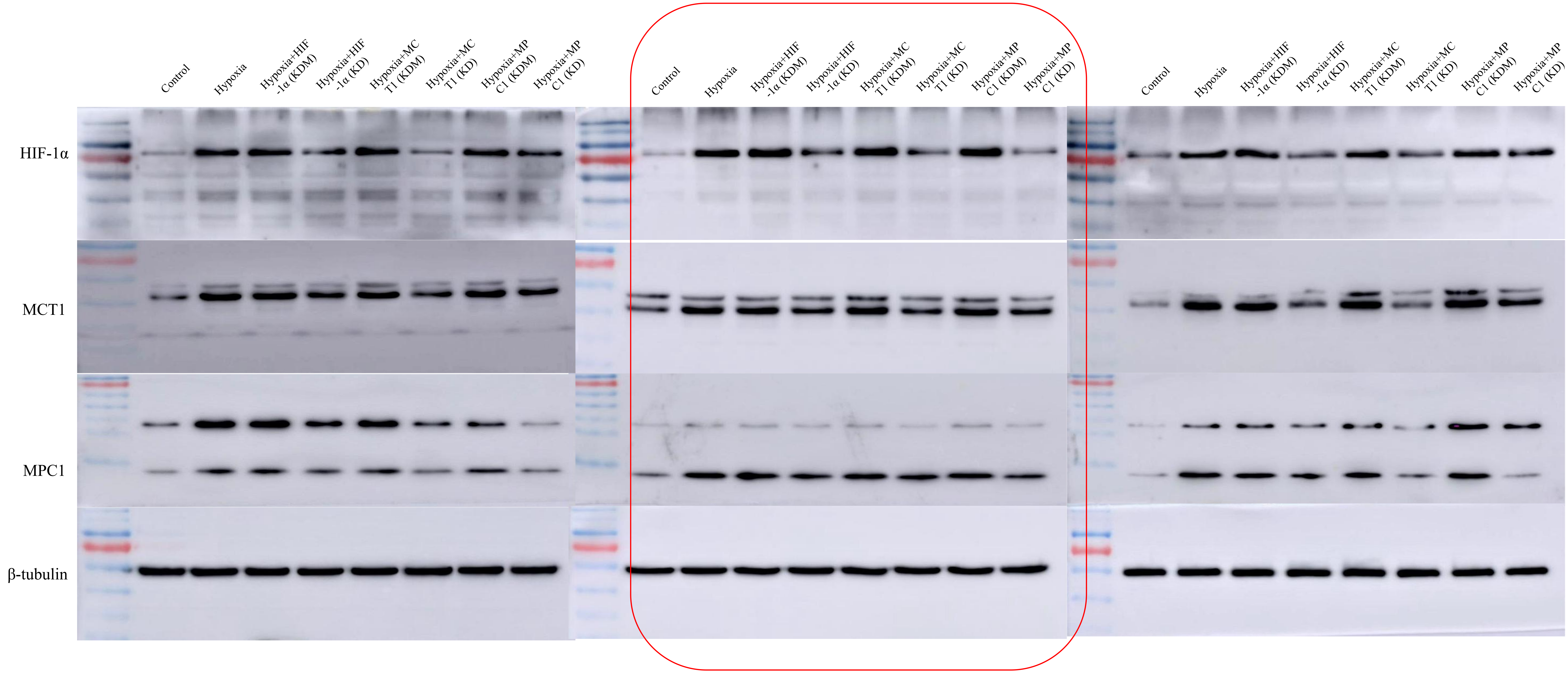

Supplement: Supplementary file 1 — Supplementary Material 1 [file 10020_2024_854_MOESM1_ESM.pdf]
